# Preface of the Special Issue on the Role of Extracellular Matrix in Development and Cancer Progression

**DOI:** 10.3390/biom12030362

**Published:** 2022-02-25

**Authors:** George Tzanakakis, Dragana Nikitovic

**Affiliations:** Laboratory of Histology-Embryology, School of Medicine, University of Crete, 71003 Heraklion, Greece

The consecutive steps of tumor growth, local invasion, intravasation, extravasation, invasion of anatomically distant sites, and immunosuppression are obligatorily perpetrated through specific interactions of the tumor cells with their microenvironment. During cancer progression, significant changes can be observed in the properties of extracellular matrix (ECM) components, which deregulate the behavior of stromal cells, promote tumor-associated angiogenesis and inflammation, and lead to the generation of a tumorigenic microenvironment. Thus, mediators originating from the ECM have a vital effect on all cellular functions implicated in cancer development and progression. ECM components, including fibrillar proteins, proteoglycans (PGs), and glycosaminoglycans (GAGs), modulate the bioavailability of active mediators, control the stiffness of the stroma immediately correlated to cancer cell invasion, and regulate the metastatic processes and angiogenesis. Various ECM-derived components can also modulate the immune response and affect the response to therapy and thus need to be taken into account when designing efficient anticancer treatment. Indeed, the ECM effectors regulate processes correlated to chemoresistance, including autophagy and apoptosis.

The Special Issue of *Biomolecules* entitled “Role of Extracellular Matrix in Development and Cancer Progression” focuses on recent findings in the structural and functional characterization of ECM components and how they relate to the processes involved in cancer pathogenesis and response to therapy. The Special Issue focusing on the crosstalk between the ECM and cellular processes features original research and review articles. These articles address several relevant topics, including HA roles in cancer, MMPs regulation of carcinoma progression, and proteoglycans mediation of cancer growth, invasion, angiogenesis, and response to therapy. Furthermore, the role of ECM effectors as diagnostic tools and targets is elaborated.

## 1. Hyaluronan in Breast Cancer

HA, the unique nonsulfated GAG, and its CD44 and RHAMM receptors have been strongly implicated in cancer progression. HA, as elaborated by Tolg et al., also has a major role in tissue injury by sequentially promoting and then suppressing inflammation and fibrosis, a duality that is featured and regulated in wound repair [1]. Tolg et al. thus focus on the hijacking of the essential response-to-injury functions of HA by tumor cells to promote their invasion and avoidance of immune detection. This is followed by a discussion of how HA metabolism is deregulated in malignant progression and how targeting HA might be used to better manage breast cancer progression.

While HA is the only GAG not normally substituted with sulfate groups, several studies suggest that sulfated hyaluronan (sHA) exhibits promising antitumor results. Koutsakis et al. show that sHA fragments attenuate breast cancer cell proliferation, migration, and invasion while increasing adhesion on collagen type I in a manner related to their estrogen receptor (ER) status [2]. Furthermore, sHA modulates the expression of epithelial-to-mesenchymal transition (EMT) markers and downregulate matrix remodeling enzymes such as the matrix metalloproteinases (MMPs). Since sHA exhibits a stronger effect on the breast cancer cell properties than the nonsulfated counterpart, a deeper understanding of the mechanism of its action could contribute to the development of novel therapeutic strategies.

## 2. MMPs Regulate Carcinoma Progression

MMPs are endopeptidases characterized by a broad range of substrate specificities and are important in ECM remodeling. However, as MMPs also have a high affinity for membrane receptors, ligands, and signaling molecules, these enzymes can be defined as cell signal regulators. In the skin, the expression of MMPs is increased in response to various stimuli, including ultraviolet (UV) radiation, one of the main factors involved in the development of basal cell carcinoma (BCC). Tampa et al. discuss the role of MMPs in the pathogenesis and evolution of BCC, as molecules involved in tumor aggressiveness and risk of recurrence, to offer an updated perspective on this field [3].

During tumor progression, elastin fragments, including a nonapeptide, AG-9, are released in the tumor microenvironment. AG-9 affects tongue squamous cell carcinoma invasive properties. Bretaudeau et al. demonstrated that AG-9 stimulates tongue squamous carcinoma cell invasion, increasing MMP-2 secretion and MT1-MMP expression [4]. The green-tea-derived polyphenol, (−)-epigallocatechin-3-gallate (EGCG), abolished AG-9-induced invasion, MMP-2 secretion, and MT1-MMP expression. The feasibility of utilizing matrix-derived signaling axes to develop novel anticancer therapeutics is underlined here. 

## 3. Proteoglycans Regulate Cancer Growth, Invasion, Angiogenesis, and Response to Therapy

Syndecans, a family of transmembrane heparan sulfate proteoglycans (HSPGs), are involved in key biological processes, such as cell proliferation, adhesion, and migration, supporting homeostasis. Still, their expression/activities are often deregulated in cancer. In addition to their roles as transmembrane PGs, syndecans’ extracellular domain can be “shedded” from the cell surface by the action of MMPS, converting them into soluble molecules capable of binding distant cell and ECM molecules. Sousa Onyeisi et al. discuss the input of the syndecan-4 member in the pathogenesis of various cancer types as its expression is commonly aberrant [5]. These authors elaborate on the point that anticancer drugs modulate syndecan-4 expression. Therefore the “take-home” line is that the syndecan-4 emerges as an important target for cancer therapy and diagnosis. 

Bertriou et al. focus on the role of syndecan-1 and -2 in the progression of pancreatic ductal adenocarcinoma (PDAC), a fatal disease with a poor prognosis [6]. Furthermore, in this review, the authors explore the potential of syndecans as therapeutic targets for this devastating disease.

Lumican, a small leucine-rich (SLRP) PGs member, is a secreted PG. Notably, this PG is involved in cellular processes associated with tumorigeneses, such as EMT, cellular proliferation, migration, invasion, and adhesion. Furthermore, lumican is expressed in various cancer tissues and is reported to have a positive or negative correlation with tumor progression. Giatagana et al. discuss the effects of lumican on cancer cell growth, invasion, motility, and metastasis, together with the repercussions on autophagy and apoptosis [7]. Finally, in light of the available data, the authors propose novel roles for lumican as a cancer prognosis marker, chemoresistance regulator, and cancer therapy target.

## 4. ECM Effectors as Diagnostic Tools and Target

In addition to cell signaling functions, the ECM molecules determine the mechanical properties of the tumor and stroma tissues. Indeed, the stroma’s mechanical properties, e.g., stiffness, are directly correlated to the pathogenesis of cancer. Ahmad et al. note that the PDAC is characterized by a dense, fibrotic stroma composed of ECM proteins that poses a significant physical barrier that is immunosuppressive and obstructs penetration of cytotoxic chemotherapy agents into the tumor microenvironment (TME) [8]. These authors discuss the significant contribution of fibrosis to the pathogenesis of pancreatic cancer, with a focus on the crosstalk between immune cells and pancreatic stellate cells that contribute to ECM deposition. Furthermore, the therapeutic strategies that target the stroma and hinder immune cell promotion of fibrogenesis, which led to mixed results, are summarized. 

Extracellular vesicles (EVs), comprising exosomes, microvesicles, and apoptotic bodies, are released by all cells into the ECM and body fluids. They play important roles in intercellular communication and matrix remodeling in various pathological conditions. Jamadi et al. characterized the tumor heterogeneity and extracellular vesicle diversity in pleural effusion exosomes as diagnostic or prognostic markers for malignant pleural mesothelioma (MPM) [9]. The corresponding ratios of mesothelin, galectin-1, osteopontin, and VEGF were higher in MPM effusions exosomes than those in the benign group. Therefore, these authors suggest that relevant diagnostic markers can be recovered from exosomes. 

## 5. Conclusions

The articles featured within this Special Issue freshly highlight the multifaceted roles of ECM molecules in cancer pathogenesis. In addition to their roles as signaling mediators, ECM molecules define the mechanical aspects of the formed tumor tissue directly correlated to the disease progression. We thank the authors for their timely contributions and hope that the “Role of Extracellular Matrix in Development and Cancer Progression” Special Issue will form an incentive for further focused research.

## Data Availability

Not applicable.
